# Normal domain temperature profile in second generation HTS tape wire

**DOI:** 10.1186/2193-1801-2-535

**Published:** 2013-10-17

**Authors:** Andrey V Malginov, Alexander Yu Kuntsevich, Vladimir A Malginov, Leonid S Fleishman

**Affiliations:** Lebedev Physical Institute RAS, 119991 Moscow, Russia; Moscow Institute of Physics and Technology (State University), 141700 Dolgoprudny, Russia; Krzhizhanovsky Power Engineering Institute, 119991 Moscow, Russia

**Keywords:** High-temperature superconductors, Phase transition, Normal zone propagation

## Abstract

**Background:**

Studies of the normal zone in high-temperature superconducting wires are extremely important for power applications, such as fault current limiters, motors, cables etc. We studied the temperature distribution and normal domain propagation in high-temperature superconducting YBCO tape with highly resistive substrate.

**Findings:**

For applied voltages exceeding a certain threshold value the normal domain was found to become unstable and started to propagate along the tape.

**Conclusions:**

The normal domain in superconducting tape with highly resistive substrate appears when voltage is applied to the sample.

At voltages greater than the threshold value, the domain starts to move.

This motion enables us to find the domain temperature and potential profile.

## Introduction

Nucleation and propagation of normal zone are of great importance for high-temperature superconductors (HTS) power applications. In previous experimental studies of the normal zone structure in HTS tapes, it was generated e.g. in vacuum by means of an external heater (Daibo et al., 2011, Pelegrin et al., 2011) or by pulsed current (Mader et al., 2011). These conditions are different from those in the HTS power devices where normal zone is generated in liquid *N*_2_ due to overcritical AC current. In the present paper we generate a normal zone by transport current in the sample. The normal zone appears to be restricted in the finite volume, i.e. we deal with normal domain (ND). Using the time resolved thermocouple and potential probe measurements we can explore the ND spatial structure.

### Methods and results

**Figure 1:**
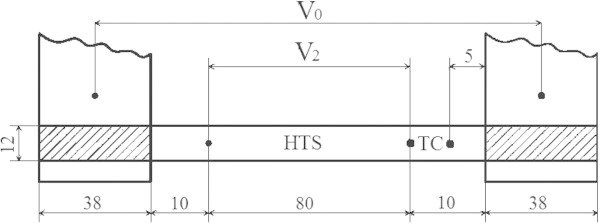
**Schematic of the sample with current leads, thermocouple, and probes (distances in mm).**

The sample of YBCO tape wire SuperPower SF12100 was tested in liquid *N*_2_. The cross-section of the 12 mm width wire has the following structure: 100 μm Hastelloy substrate, 1 μm YBCO, 1.5 μm Ag. Both nominal and measured critical currents of the wire are about 300 A, critical temperature is about 91K. Figure 1 shows the geometry of the sample with the thermocouple (TC) and potential probes.

The procedure of our AC-measurements is described in detail in Ref. (Fleishman et al., 2010). A constant amplitude 50 Hz AC voltage *V*_0_ is applied across the current leads to the HTS sample at the moment *t*_0_.

Then, current through the sample *I*, temperature of the TC *T*, and voltage in the middle part of the sample *V*_2_ are being measured with 1 ms time resolution within 40 s interval. ND is generated by local overcritical current Joule heating in a specific weak tape segment due to sample inhomogeneity. Location of this weak place (5 mm from the right current lead) was determined by eye from liqud nitrogen boiling in the preliminary experiment. TC was soldered near this point.

**Figure 2:**
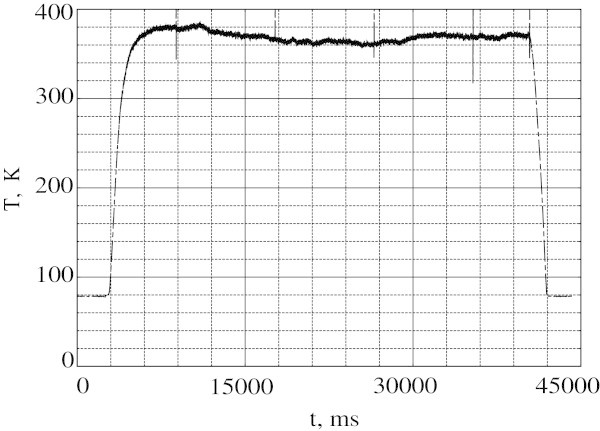
**Temperature as a function of time at** ***V***_**0**_ **= 0.76 V.**

Figure 2 shows a typical temperature *T* versus time plot after an application of *V*_0_ = 0.76 V AC voltage. The data indicate that the temperature achieves its maximum in 3 s and almost does not change until switching the voltage off. During this period the voltage *V*_2_ between the potential probes is approximately zero.

The data in the range 0.3 V < *V*_0_ < 0.76 V demonstrate qualitatively similar behavior: once normal domain is formed it stays in the same place and its parameters remain unchanged. It means that for *V*_0_ < 0.76 V the normal zone is located between the potential probe and the right current lead, and TC measures the ND temperature.

**Figure 3:**
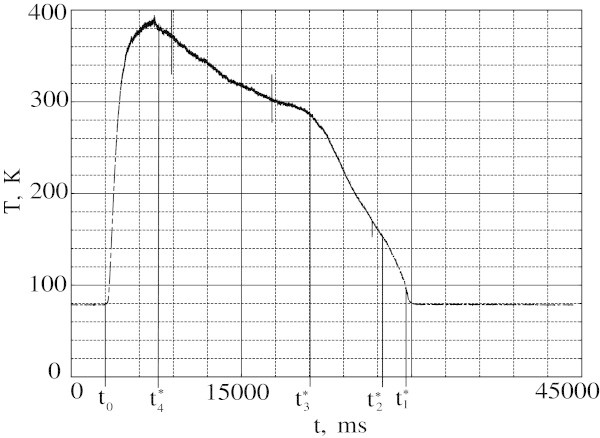
**Temperature as a function of time at** ***V***_**0**_ **= 0.79 V.**

**Figure 4:**
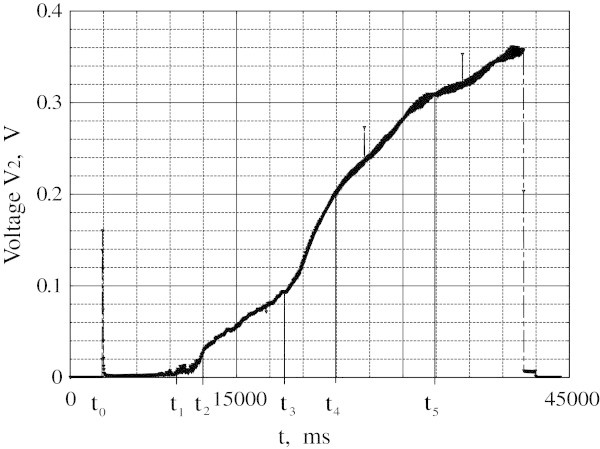
**The amplitude value of voltage** ***V***_**2**_ **across the middle part of the sample as a function of time for** ***V***_**0**_ **= 0.79V.**

The temperature versus time dependence changes drastically when the voltage step *V*_0_ is increased to 0.79 V (Figure 3). The temperature reaches almost the same maximum value as for lower *V*_0_ value but instead of being constant decreases afterwards. The rate of temperature drop changes at points *t*_4_^*^, *t*_3_^*^, *t*_2_^*^, *t*_1_^*^. At the same time the voltage *V*_2_≠*0* appears between the potential probes (Figure 4) exhibiting irregularities at points *t*_*1*_*, t*_*2*_*, t*_*3*_*, t*_*4*_. The data in Figures 3 and 4 suggest that ND begins to move as a whole from the sample end towards its center. The irregularities in the *T(t)* and *V*_2_*(t)* dependences correspond to the motion of specific ND regions across the TC and voltage probe respectively. TC traces the outgoing right edge of the domain. Passing of the left edge is traced by *V*_2_, e.g. from t_1_ to t_2_ a foremost portion crosses a probe. This portion is cooled by film boiling and has temperature slightly above 77K. From t_2_ to t_3_ the left edge moves into the segment between the potential probes. From t_3_ to t_5_ the ND top part passes the probe and the temperature is maximal at t_4_. The interval from t_4_ to t_5_ corresponds to the TC measurement interval from t_4_^*^ to t_3_^*^. At the period from t_3_^*^ to t_2_^*^ the right edge passes TC and after t_2_^*^ the outermost portion crosses TC position. All the processes occur at the voltage of *V*_0_ = 0.79 V. Current achieves a stable *I* = 50 A level in 3 s after *t*_0_. The ND velocity estimated from the rate of the resistance change is about 1 mm/s. To obtain temperature and potential profiles of the ND as function of distance one should multiply *t*-axis in Figures 3 and 4 by scale factor 1 mm/s. For higher *V*_0_ values ND also drifts towards sample center and both *T(t)* and *V*_2_*(t)* dependencies demonstrate the similar behavior.

**Figure 5:**
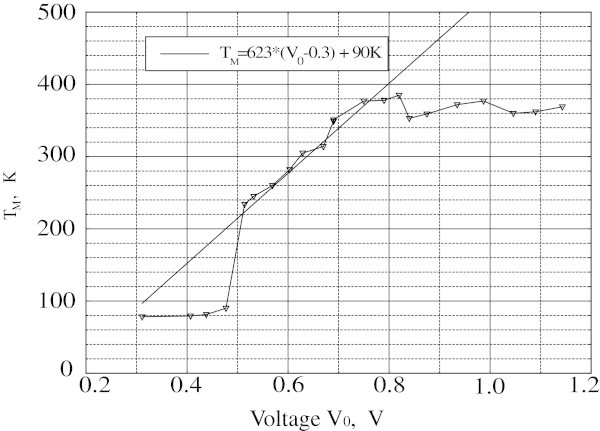
**Maximum temperature as a function of amplitude** ***V***_**0.**_

Figure 5 shows the maximum temperature measured by TC as a function of *V*_0_ value. When *V*_0_ < 0.3 V the ND is so small that TC is out of it and does not respond to heating.

We have shown that for voltages greater than 0.79 V the domain moves and the TC measures its temperature profile. In the interval 0.3 V < *V*_0_ < 0.79 V the domain stays within the weak place and TC measures the temperature near its top. In this case the ND maximum temperature *T*_M_ (in K) as a function of applied voltage *V*_0_ (in V) is approximated by: *T*_M_ = 623 (*V*_0_-0.3) + 90. This fit allows one to estimate the upper boundary of the voltage (*V* ~ 1 V), for which the tape survives (*T* ~ 500 K).

## Conclusions

To summarize, the above experimental data indicate that the normal domain in superconducting tape with highly resistive substrate appears when voltage is applied to the sample. At voltages greater than 0.79 V the domain starts to move. This motion enables us to find the domain temperature and potential profile from the simultaneous measurements with a thermocouple and potential probes.
